# Correction: Arshad et al. A Comparative Modelling Study of New Robust Packaging Technology 1 mm^2^ VCSEL Packages and Their Mechanical Stress Properties. *Micromachines* 2022, *13*, 1513

**DOI:** 10.3390/mi16040390

**Published:** 2025-03-28

**Authors:** Khairul Mohd Arshad, Muhamad Mat Noor, Asrulnizam Abd Manaf, Hiroshi Kawarada, Shaili Falina, Mohd Syamsul

**Affiliations:** 1 Institute of Nano Optoelectronics Research and Technology (INOR), Universiti Sains Malaysia, Sains@USM, Bayan Lepas 11900, Pulau Pinang, Malaysia; khairulmohdarshad@student.usm.my; 2 Faculty of Mechanical and Automotive Engineering Technology, Universiti Malaysia Pahang, Pekan 26600, Pahang, Malaysia; muhamad@ump.edu.my; 3 Collaborative Microelectronic Design Excellence Centre (CEDEC), Universiti Sains Malaysia, Sains@USM, Bayan Lepas 11900, Pulau Pinang, Malaysia; eeasrulnizam@usm.my (A.A.M.); shailifalina@usm.my (S.F.); 4 Faculty of Science and Engineering, Waseda University, Shinjuku, Tokyo 169-8555, Japan; kawarada@waseda.jp; 5 The Kagami Memorial Laboratory for Materials Science and Technology, Waseda University, 2-8-26 Nishiwaseda, Shinjuku, Tokyo 169-0051, Japan

## Error in Figure 1a,b

In the original publication [1], there was a mistake in ‘**Figure 1.** The VCSEL package. (**a**) component structure for package; (**b**) the actual package with diffuser and SEM image for microstructure on top lens’, as published. ‘Should be just share the illustration image for package and microstructure lens on top of lens’. The corrected ‘**Figure 1.** (**a**) The illustration of VCSEL package; (**b**) the illustration of microstructure of lens on top of VCSEL package’ appears below.

**Figure 1:**
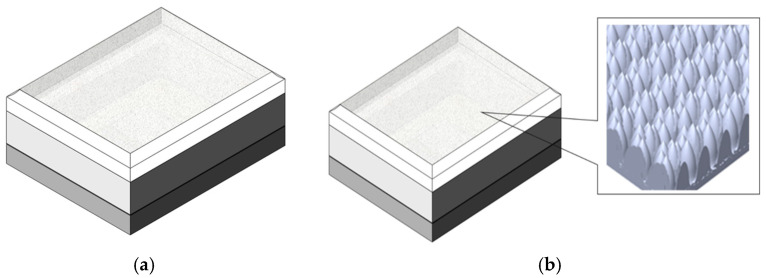
(**a**) The illustration of VCSEL package; (**b**) the illustration of microstructure of lens on top of VCSEL package.

## Error in Figure 2

In the original publication [1], there was a mistake in ‘**Figure 2.** The convex share structure was developed for the microstructure of the diffuser on the top of a lens’, as published. ‘Should be just share the illustration image microstructure lens on top of lens’. The corrected ‘**Figure 2.** The illustration of the microstructure of the lens on top of the VCSEL package’ appears below.

**Figure 2:**
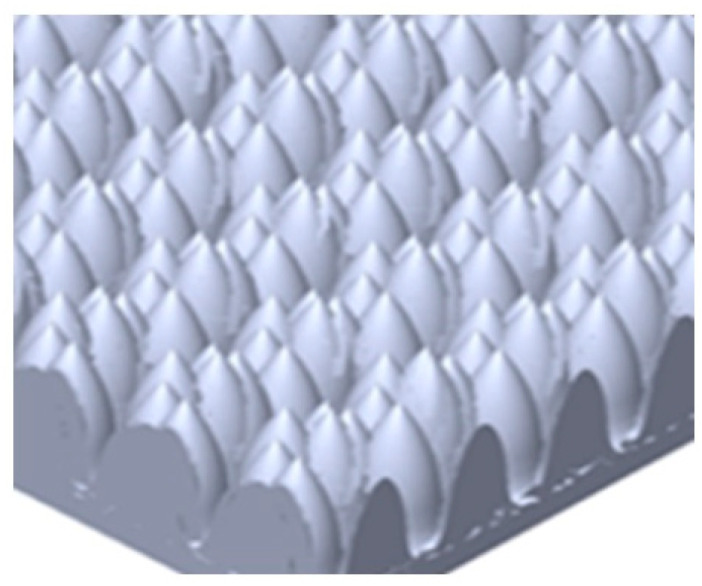
The illustration of the microstructure of the lens on top of the VCSEL package.

## Error in Figure 3

In the original publication [1], there was a mistake in ‘**Figure 3.** (**a**) Top view optical image of the cracked lens unit of VCSEL; (**b**) cross section view optical images of cracks observed in different positions labeled A–E; and (**c**) SEM images of respective positions’, as published. ‘Should be just share the illustration of microstructure lens have crack and damage’. The corrected ‘**Figure 3.** (**a**,**b**) The illustration of microstructure has crack and damage of microstructure lens’ appears below.

**Figure 3:**
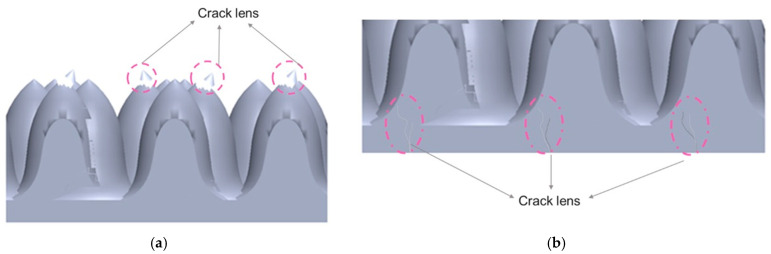
(**a**,**b**) The illustration of microstructure has crack and damage of microstructure lens.

The authors state that the scientific conclusions are unaffected. This correction was approved by the Academic Editor. The original publication has also been updated.

## References

[1] Arshad K.M., Noor M.M., Manaf A.A., Kawarada H., Falina S., Syamsul M. (2022). A Comparative Modelling Study of New Robust Packaging Technology 1 mm2 VCSEL Packages and Their Mechanical Stress Properties. Micromachines.

